# Immune complex induced arthritis in rats: role of lipid mediators on cell infiltration

**DOI:** 10.1155/S0962935196000178

**Published:** 1996-04

**Authors:** F. A. C. Rocha, L. E. C. Andrade, S. Jancar

**Affiliations:** 1 Universidade Federal do Ceará Rua Tiburcio Cavalcante 2100/1201 CEP 60125-101 Fortaleza CE Brazil; 2 Universidade Paulista de Medicina SP Brazil; 3 Universidade de São Paulo SP Brazil

## Abstract

We investigated the participation of lipid mediators in an experimental immune complex (IC) arthritis model in rats. The animals were subjected to intraarticular injection of anti-bovine sertLm albumin (BSA) IgG antibodies followed by i.v. injection of BSA. Histopathological analysis of the synovial membranes disclosed infiltration of polymorphonuclear (PMN) cells and vascular congestion. Slight increase in vascular permeability, measured by Evans blue dye extravasation into the joints, was detected after 3 h of arthritis. Cellular influx into the articular cavities was most evident at the sixth hour of arthritis with predominance of PMN. Pretreatment with either indomethacin, a cyclooxygenase inhibitor, or L-660,711, a peptido-leukotriene antagonist, did not inhibit cell infiltration, whereas pretreatment with either L-663,536, a 5-lipoxygenase inhibitor, or L-655,240, a thromboxane antagonist, significantly inhibited the phenomenon. Pretreatment with WEB 2170, a platelet activating factor (PAF) antagonist, also significantly inhibited cell influx. These results suggest that thromboxane, LTB_4_ and PAF mediate cell infiltration in this IC arthritis model.


Mediators of Inflammation 5, 104–109 (1996)

### Immune complex induced arthritis in rats: role of lipid mediators on cell infiltration

F. A. C. Rocha,[1,CA] L. E. C. Andrade[2] and S. Jancar[3]

[1]Universidade Federal do Ceará, Rua Tiburcio Cavalcante, 2100/1201, CEP 60125-101, Fortaleza, CE, Brazil. Fax: (+55) 85 2439333; [2]Universidade Paulista de Medicina, SP, Brazil; and [3]Universidade de São Paulo, SP, Brazil

[CA]Corresponding Author

**We investigated the participation of lipid mediators in an experimental immune complex (IC) arthritis model in rats. The animals were subjected to intraarticular injection of anti-bovine serum albumin (BSA) IgG antibodies followed by i.v. injection of BSA. Histopathological analysis of the synovial membranes disclosed infiltration of polymorphonuclear (PMN) cells and vascular congestion. Slight increase in vascular permeability, measured by Evans blue dye extravasation into the joints, was detected after 3 h of arthritis. Cellular influx into the articular cavities was most evident at the sixth hour of arthritis with predominance of PMN. Pretreatment with either indomethacin, a cyclooxygenase inhibitor, or L-660,711, a peptido-leukotriene antagonist, did not inhibit cell infiltration, whereas pretreatment with either L-663,536, a 5-lipoxygenase inhibitor, or L-655,240, a thromboxane antagonist, significantly inhibited the phenomenon. Pretreatment with WEB 2170, a platelet activating factor (PAF) antagonist, also significantly inhibited cell influx. These results suggest that thromboxane, $LTB_4$ and PAF mediate cell infiltration in this IC arthritis model.**

**Key words**: Arthritis, Arthus reaction, eicosanoids, platelet activating factor

### Introduction

Immune complex (IC) deposition is an important event in the pathogenesis of rheumatoid arthritis (RA). This assumption is supported by the detection of IC deposits in the synovium and articular cartilage, as well as by the possibility of rheumatoid factor self-aggregation in large insoluble immune complexes and the association of circulating immune complexes with disease severity in some RA patients.[1] The most commonly used experimental models of RA, the antigen-induced arthritis and the adjuvant-induced arthritis, exhibit close resemblance to the pathophysiology of RA. However, they do not allow a precise investigation of the isolated contribution of IC to the development of arthritis.

Induction of a reverse passive Arthus reaction in rabbit knee joints promotes a modest increase in vascular permeability, pronounced cellular infiltration, mostly polymorphonuclear (PMN) neutrophils, and cartilage destruction, being considered as a suitable experimental model to study the mechanisms involved in articular lesions dependent on IC formation.[2] Although the recruitment and activation of these cells in IC-induced lesions is considered essential to the full-blown development of the reaction,[3] the factors governing these phenomena are still obscure.

Previous data have shown that eicosanoids and platelet activating factor (PAF) are involved in IC disease models. These lipid mediators can alter vascular permeability and microcirculatory blood flow. In addition, they promote cell migration and activation, causing release of other inflammatory mediators as well as reactive oxygen intermediates and lysosomal enzymes, which are effectors of local tissue damage. Detection of prostaglandin $E_2$, leukotriene $B_4$ and thromboxane $B_2$ in the bronchoalveolar lavage fluid[4] and peritoneal exudate[5] in Arthus reactions in rats argues for the participation of these substances in IC-induced lesions. Moreover, lung haemorrhagic lesions induced by IC were shown to be mediated by PAF and $LTB_4$.[6] However, the role of these mediators in IC-induced arthritis has not been established yet.

The aim of the present study was to characterize the early events of an arthritis caused exclusively by immune complexes. To this purpose, a reverse passive Arthus reaction was induced in the knee joint of rats. Synovial histopathological alterations, vascular permeability, and cell influx into the joints were evaluated. The contribution

© 1996 Rapid Science Publishers of PAF and eicosanoids to cell infiltration into the joints was also investigated.

### Materials and Methods

*Animals:* Male Wistar rats (250–350 g) from our own animal facilities were used throughout the experiments.

*Induction of passive reverse Arthus reaction in the knee joints:* Rats were anaesthetized by i.p. injection of chloral hydrate (400 mg/kg). Rabbit IgG antibodies to BSA (200 μg in 0.1 ml saline) were injected into the knee joints, followed by i.v. injection of 1 mg BSA in 0.2 ml saline. Control animals received the same amount of the antibody intraarticularly followed by saline injection i.v.

*Collection of synovial exudate:* The animals were anaesthetized and killed by cervical dislocation and exsanguinated. The synovial cavity of the knee joints was then washed with 0.4 ml saline containing 5 U/ml heparin. The exudates were collected by aspiration, centrifuged (500 × ***g*** for 10 min) and the cell-free supernatant stored at –20°C for further analysis.

*Evaluation of increase in vascular permeability:* Groups of animals received i.v. injection of Evans blue dye (20 mg/kg), dissolved in saline, 15 min prior to arthritis induction. The animals were sacrificed at different times and the synovial exudate collected for determination of the amount of extravasated dye. The concentration of the dye was determined colorimetrically at 630 nm and compared to a standard curve. Results were expressed as μg of Evans blue per ml of exudate.

*Histopathological analysis and immunofluorescence technique:* Synovial tissue sections were processed for routine haematoxylin–eosin staining and also for direct immunofluorescence. Briefly, 3 μm thick cryosections of synovial tissues were mounted on glass slides, fixed in acetone at 20°C for 8 min, and air-dried. Tissue sections were incubated with FITC-labelled goat anti-rabbit IgG or FITC-labelled goat anti-human IgG diluted to 1:100 in PBS (0.1 M NaCl, pH 7.2) for 30 min at 37°C in wet chamber. After washing twice in PBS for 10 min, slides were mounted in glycerine at pH 9.0 and observed by using fluorescence microscopy.

*Cell counting:* The synovial exudates were centrifuged (500 × ***g*** for 10 min) and the cell pellet resuspended in 0.1 ml saline. Total cell counts were determined using a Neubauer chamber. Differential counts were performed on fixed and stained cell suspensions (0.05% crystal violet dissolved in 30% acetic acid).

*Drug treatments:* Groups of animals were treated with the following drugs 30 min prior to arthritis induction: indomethacin (4 mg/kg, i.v.); WEB 2170 (5 mg/kg, i.v.); L-660,711 (10 mg/kg, i.v.); L-655,240 (5 mg/kg, i.v.). The compound L-663,536 (10 mg/kg, *per os*), was injected 20 h prior to arthritis.

*Drugs and reagents:* The compounds L-663,536, L-660,711, and L-655,240 were kindly supplied by Merck Frosst Lab., Canada, and WEB 2170 by Boehringer–Ingelheim, Germany. Indomethacin, BSA, rabbit IgG anti-BSA antibodies, and FITC-labelled goat anti-rabbit IgG were purchased from Sigma Chem Co., USA, and FITC-labelled goat anti-human IgG from Hemagen, USA.

*Statistical analysis:* ANOVA or Student's *t*-test were used to compare means. $p < 0.05$ was considered significant.

### Results

An Arthus reaction was induced by injection of rabbit anti-BSA IgG antibodies into the articular cavity of rats, followed by i.v. injection of the antigen (IC group). The control group received an injection of the antibody into the articular cavity and saline i.v.

*Histopathological analysis:* Figure 1(A) illustrates a synovium specimen obtained 6 h after arthritis induction, showing interstitial haemorrhage and infiltration of PMN cells. The control groups exhibited none of these alterations (not shown). The direct immunofluorescence, using FITC anti-rabbit IgG, aspect of a synovium specimen from a rat subjected to the Arthus reaction is depicted in Fig. 1(B), showing granular IC deposits which were not seen in the control group (not shown). A direct immunofluorescence using anti-human IgG conjugate did not show fluorescence staining, thus ensuring the specificity of the anti-rabbit IgG conjugate used (data not shown).

*Increase in vascular permeability:* The alteration in vascular permeability, measured as the concentration of Evans blue dye extravasated into the joint cavity was evaluated 10 min, 30 min, 1 h, and 3 h after arthritis induction. A significant ($p < 0.05$) increase in extravasation of the dye was observed only in the 3 h exudates, being 0.59 μg/

A

B

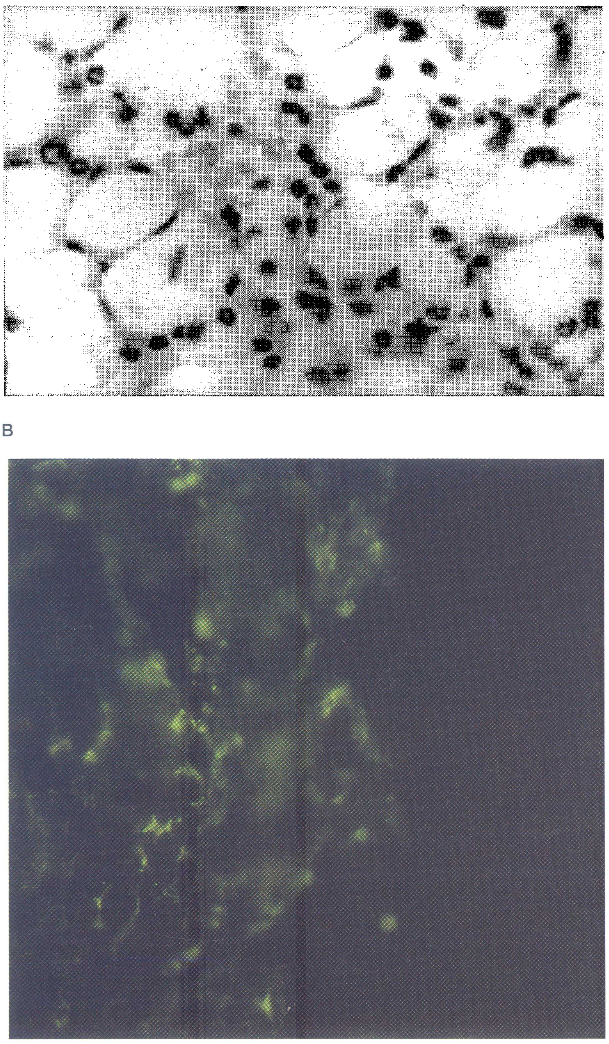

FIG. 1. (A) Synovial section from rats 6 h after induction of immune complex arthritis, showing interstitial haemorrhage and polymorphonuclear cell inflammatory infiltrate. (Haematoxylin-eosin; original magnification × 400.) (B) Direct immunofluorescence of synovial section of rats 6 h after induction of IC arthritis, showing granular IC deposits, which were not seen in the control group (original magnification × 400).

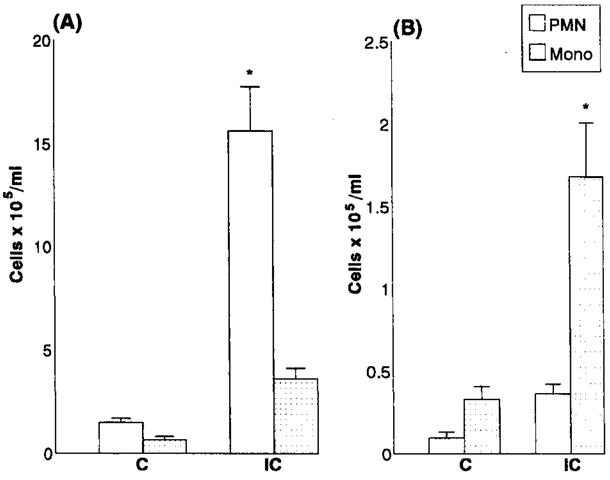

FIG. 2. Polymorphonuclear and mononuclear cell counts in the synovial cavity of rats 6 h (A) or 24 h (B) after induction of IC arthritis (IC). Control groups (C) received the antibodies intraarticularly and saline i.v. Data represent the mean ± S.E.M. ($n$ = 6–8 animals). *$p < 0.05$ as compared to the control group using Student's $t$-test.

ml ($n$ = 8), compared with 0.21 μg/ml ($n$ = 5) in the control group.

*Cellular influx into the knee joints:* Figure 2 shows the cell counts in the synovial exudates of the IC and control groups after 6 h (A) and 24 h (B) of arthritis induction. After 6 h of arthritis, the total number of leukocytes was about nine times greater in the IC group (1924 ± 264.66 × $10^3$ cells/ml) compared with the control group (213.32 ± 37.56 × $10^3$ cells/ml), with a predominance of PMN cells. After 24 h of arthritis, there was still a statistically significant difference between the two groups although, at this time, the difference was due to a five times greater number of mononuclear cells in the IC group.

*Role of lipid mediators on the cellular influx:* In order to investigate the role of eicosanoids and PAF in the cell influx into the knee joints, we studied the effect of treating the animals with indomethacin (a cyclooxygenase inhibitor), L-663,536 (a lipoxygenase inhibitor), L-655,240 (a thromboxane antagonist), L-660,711 (a peptido-leukotrienes antagonist) or WEB 2170 (a PAF antagonist) prior to arthritis induction. Figure 3 shows that pretreatment with either indomethacin or L-660,711 did not alter the number of cells present in the exudate collected 6 h after induction of the reaction. Pretreatment with either L-655,240, WEB 2170 or L-663,536 caused a decrease of 77%, 69%, and 54.7% in cell counts, respectively. The efficacy of the inhibitors indomethacin and L-663,536 was confirmed by using ELISA to evaluate their effect on $PGE_2$ and $LTB_4$ release into the synovial exudates. Indomethacin completely blocked $PGE_2$ release whereas L-663,536 caused an 80% reduction of $LTB_4$ release (data not shown). These results suggest that thromboxane, PAF and leukotriene $B_4$ are involved in cell migration in this IC arthritis model.

### Discussion

In this IC arthritis model, the groups subjected to the reverse passive Arthus reaction were always compared to the group that received the antibody intraarticularly, but not the antigen. Since the mere administration of antibody into the joints may result in a mild synovitis, the comparison of both groups was essential to allow

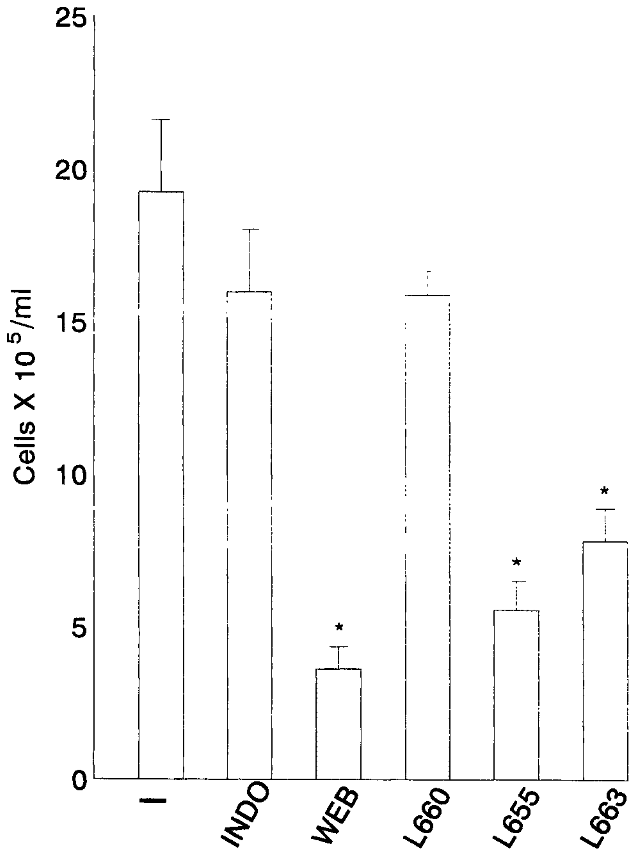

FIG. 3. Effect of treatment on the cellular influx into the synovial cavity of rats, 6 h after induction of IC arthritis. The first group received no treatment and the subsequent ones were pretreated with indomethacin (4 mg/kg i.v., $n = 5$), WEB 2170 (5 mg/kg i.v., $n = 9$), L-660,711 (10 mg/kg i.v., $n = 4$), L-655,240 (5 mg/kg i.v., $n = 6$), L-663,536 (10 mg/kg *per os*, $n = 7$). Data represent the mean ± S.E.M. $^*p < 0.05$ as compared to the control group, using one-way ANOVA.

the study of the inflammatory process induced solely by the deposition of immune complexes.

The histopathological analysis revealed vascular congestion and cellular infiltrate, composed mainly of neutrophils at 6 h and mononuclear cells at 24 h. Similar findings were reported in rabbits subjected to IC arthritis.[2] The demonstration of granular immunofluorescent deposits in the synovia of the rats subjected to the Arthus reaction, but not in the control group, provides a strong indication that the inflammatory changes were mostly caused by IC deposition in the synovial tissue.

The increase in vascular permeability as measured by the analysis of the concentration of Evans blue dye extravasated to the synovial cavity was significant only when measured over a period of 3 h following induction of the reaction.

These results differ from those obtained in a model of active Arthus reaction in rabbits, where a significant increase in vascular permeability was observed.[7] Since active immunization induces production of precipitating antibodies as well as antibodies cytophylic for mast cells, the latter would promote release of mast cell derived mediators which would account for the increased vasopermeability. In the present study, we used rabbit IgG antibodies which are not cytophylic for rat mast cells, in order to ensure a reaction mediated predominantly by immune complexes. In this case, increase in vascular permeability is not a prominent event, as we have previously reported in IC-induced alveolitis.[6] One possible speculation is that, in this type of reaction, there is a release of vasoconstricting substances which may limit plasma extravasation. In support of this assumption, it was shown that release of slow reacting substance of anaphylaxis (SRS-A) in passive Arthus reaction markedly inhibited carrageenin oedema.[8]

The infiltration of inflammatory cells into the synovial cavity peaked 6 h after arthritis induction, with a predominance of PMN cells, and declined after 24 h, when mononuclear cells became predominant. These results are in accordance with previous data showing leukocyte infiltration in the reverse passive Arthus reaction in rabbit joints[2] as well as in the antigen-induced arthritis model.[7,9]

Pretreatment with specific inhibitors or antagonists of eicosanoids and PAF showed that inhibition of leukotriene synthesis by compound L-663,536,[10] as well as antagonism of thromboxane by L-655,240[11] or of PAF by WEB 2170[12] inhibited cellular influx into the synovial cavities. Inhibition of cyclooxygenase by indomethacin or antagonism of peptido-leukotrienes by compound L-660,711[13] did not affect cell influx.

That the concentrations of the inhibitors used in this study were adequate was confirmed by the reduction of $LTB_4$ and $PGE_2$ levels in the synovial exudates by L-663,536 and indomethacin, respectively. The concentration of the PAF antagonist, WEB 2170, was assayed against PAF-induced vasopermeability in rats, being 5 mg/kg—the dose that provoked the best inhibition. A recent publication from our group demonstrates the efficacy of this dose on PAF-induced vascular permeability in rat lungs.[14] The concentration of L-655,240 was taken from a relevant published report where this compound selectively antagonized the effects of a thromboxane analogue both *in vivo* and *in vitro*.[11] The peptido-leukotriene antagonist L-660,711 was used at doses ten times higher than those reported to inhibit antigen-induced bronchoconstriction in sensitized rats.[13]

The fact that an inhibitor of 5-lipoxygenase inhibited cell influx whereas an antagonist of peptido-leukotrienes had no effect suggests that leukotriene $B_4$ is responsible for this phenomenon. Taken together, these results suggest that $LTB_4$, thromboxane, and PAF contribute to cell infiltration in IC arthritis.

The presence of $LTB_4$ in the synovial fluid of arthritis patients has already been demonstrated.[15] Moreover, neutrophils and macrophages from RA patients stimulated with calcium ionophore A23187 release more $LTB_4$ than do those of healthy volunteers.[16] Besides promoting PMN adhesion to vascular endothelial cells,[17] $LTB_4$ is also a potent chemoattractant to both neutrophils and macrophages.[18] However, few studies have focused on the importance of leukotrienes in arthritis. To our knowledge, this is the first demonstration that $LTB_4$ contributes to leukocyte infiltration in arthritis.

Non-steroidal anti-inflammatory drugs (NSAIDs) are the most commonly prescribed substances to treat RA patients. It is well known that these drugs provide only symptomatic relief to RA patients, without altering the disease outcome. This could be due to the cessation of an inhibitory effect of the cyclooxygenase products on leukotrienes synthesis[19] or by a preferential shift of the substrate pool into the lipoxygenase pathway, thus enhancing leukotriene production.[20] This putative increase in leukotriene release could partially explain the lack of efficacy of NSAIDs in modifying the natural course of RA.

Previous data have shown that thromboxane $A_2$ is chemotactic to mouse neutrophils *in vitro*.[21] Additionally, econazole, an imidazole compound which inhibits thromboxane synthesis, inhibited cell infiltration in carrageenin pleurisy.[22] This effect of thromboxane could be explained by its ability to enhance neutrophil adhesion to endothelial cells, via activation of the CD18 receptor on neutrophils.[23] In our experiments, the thromboxane antagonist significantly inhibited cell influx, suggesting that thromboxane is involved in cell migration in arthritis. We have previously demonstrated that thromboxane is involved in PAF-induced vasoconstriction in the synovial microcirculation of normal rabbit joints.[24] Our present data indicating the participation of $LTB_4$ and $TXB_2$ in the cell infiltration in this IC arthritis model suggest that these lipid mediators may have a more important regulatory role on inflammatory arthropathies than has previously been ascribed to them.

Our results also showed that pretreatment of the animals with WEB 2170, a specific PAF antagonist, significantly reduced cell infiltration, thus implicating PAF in this phenomenon. The potential importance of PAF in arthritis can be illustrated by its detection in the synovial fluid of experimental models of arthritis.[25] Furthermore, it was reported that PAF contributes to cell migration in the antigen-induced arthritis model in rabbits, an effect that could result from either a direct action of PAF or an indirect one via the release of other inflammatory mediator(s).[26] We have recently observed that the PAF antagonist WEB 2170, but not indomethacin, L-663,536 or L-655,240 inhibited TNF release in this IC arthritis model (Rocha *et al.*, submitted). These results suggest that the effect of PAF on cell migration might be through TNF release. It also points to interesting interactions between lipid mediators and cytokines. Another mechanism that might contribute to cell migration is related to complement activation. However, we believe that C5 fragments are not essential for cell migration in IC reactions, based on previous observations that the temporal profile of neutrophil accumulation in response to IC deposition is similar in coisogenic strains of mice, where one of them is genetically deficient in the C5 component.[27]

The exact role of neutrophils in the pathogenesis of arthritis is a subject of debate. Although it seems indisputable that these cells are responsible for the release of inflammatory mediators, lysosomal enzymes and reactive oxygen species, a direct role for them in mediating cartilage breakdown is not clearly established. In an IC arthritis model in mice it was suggested that these cells are involved in cartilage destruction.[28] However, in the antigen-induced arthritis model in rabbits, it was suggested that neutrophils might not be essential for cartilage degradation, since the animals still presented cartilage breakdown after being rendered neutropenic by pretreatment with nitrogen mustard.[9] More recently, in an *in vitro* study, it was demonstrated that neutrophils were able to degrade both proteoglycans and collagen in human cartilage, an effect that was facilitated by previous stimulation of the neutrophils with immunoglobulins.[29] Such data are corroborated by previous studies suggesting that immunuglobulins might facilitate neutrophil adhesion to cartilage surface, where these cells could then be activated.[30] Another explanation could be that activation of the Fc receptor of previously primed neutrophils might potentiate the release of inflammatory mediators, as well as release of reactive oxygen species and lysosomal enzymes by these cells, thus resulting in increased tissue damage.

Since IC deposition is involved in some rheumatic conditions, including rheumatoid arthritis, systemic lupus erythematosus and vasculitis, the possibility of studying the mechanisms governing IC-induced lesions in joints makes this model relevant to the understanding of the pathophysiology of synovitis.

The IC arthritis model described here proved to be a suitable and reproducible model of experimental arthritis. The participation of eicosanoids, mainly leukotrienes and thromboxane, as well as PAF in the regulation of cell migration described here suggests that inhibitors or antagonists of these substances might be valuable alternatives to the therapy for inflammatory arthropathies.

### References

1. Harris Jr ED. Rheumatoid arthritis: the clinical spectrum. In: Kelley WN, Harris Jr ED, Ruddy S, Sledge CB, eds. *Textbook of Rheumatology*. Philadelphia: W.B. Saunders Company, 1985; 915–949.
2. Foster SJ, Cunliffe CJ, McCormick ME. Polymorphonuclear leukocytes induce damage to the articular cartilage in acute immunologic arthritis in rabbits. *Biochem Pharmacol* 1988; **37**: 1181–1183.
3. Johnson JK, Ward PA. Acute immunologic pulmonary alveolitis. *J Clin Invest* 1974; **54**: 349–357.
4. Tavares de Lima W, Teixeira CFP, Sirois P, Jancar S. Involvement of eicosanoids and PAF in immune complex alveolitis. *Braz J Med Biol Res* 1989; **22**: 745–748.
5. Jancar S, De Giacobbi G, Mariano M, Sirois P, Braquet P. Immune-complex induced pancreatitis effect of BN-52021 a selective PAF-antagonist. *Prostaglandins* 1988; **35**: 757–770.
6. Tavares de Lima W, Sirois P, Jancar S. Immune-complex alveolitis in the rat: evidence for platelet activating factor and leukotrienes as mediators of the vascular lesions. *Eur J Pharmacol* 1992; **213**: 63–70.
7. De Brum-Fernandes AJ, de Falco V, Rocha FAC, Jancar S. Paradoxical effect of dexamethasone administration to rabbits with antigen-induced arthritis. *Inflammopharmacol* 1992; **1**: 289–294.
8. Jancar S, Faccioli LH. The anti-edematogenic effect of SRS as an additional factor in the mode of action of non-steroid anti-inflammatory drugs. *Eur J Pharmacol* 1985; **112**: 153–160.
9. Pettipher ER, Henderson B, Moncada S, Higgs GA. Leucocyte infiltration and cartilage proteoglycan loss in immune arthritis in the rabbit. *Br J Pharmacol* 1988; **95**: 169–176.
10. Gillard J, Ford-Hutchinson AW, Chan C *et al.* L-663,536 (MK-886) (3-[1-(4-chlorobenzyl)-3-t-butyl-thio-5-isopropylindol-2-yl]-2,2-dimethylpropanoic acid), a novel, orally active leukotriene biosynthesis inhibitor. *Can J Phys Pharmacol* 1989; **67**: 456–464.
11. Hall RA, Gillard J, Guindon Y, Letts G, *et al.* Pharmacology of L-655240 (3-[1-(4-chlorobenzyl)-5-fluoro-3-methyl-indol-2-yl]2,2-dimethylpropanoic acid): a potent, selective thromboxane/prostaglandin endoperoxide antagonist. *Eur J Pharmacol* 1987; **135**: 193–201.
12. Heuer H, Casals-Stenzel J, Muacevic G, *et al.* Effect of WEB 2170 and STY 2108, two hetrazepines with antagonistic activity on PAF-induced changes *in vivo* and *in vitro*. *Clin Exp Pharmacol Physiol* 1988; Suppl **13**: 7–13.
13. Jones TR, Zamboni R, Belley M, *et al.* Pharmacology of L-660,711 (MK-571): a novel potent and selective leukotriene D4 antagonist. *Can J Physiol Pharmacol* 1989; **67**: 17–28.
14. Tavares de Lima W, Kwasniewski FH, Sirois P, Jancar S. Studies on the mechanisms of PAF induced vasopermeability in rat lungs. *Prostaglandins, Leukotrienes, Essent Fatty Acids* 1995; **52**: 245–249.
15. Klickstein LB, Shapleigh C, Goetzl EJ. Lipoxigenation of arachidonic acid as a source of polymorphonuclear chemotactic factors in synovial fluid and tissue in rheumatoid arthritis and spondyloarthritis. *J Clin Invest* 1980; **66**: 1166–1170.
16. Belch JJF, O'Dowd A, Ansell D, Sturrock RD. Leukotriene B4 production by peripheral blood neutrophils in rheumatoid arthritis. *Scand J Rheumatol* 1989; **18**: 213–219.
17. Gimbrone MA, Brock AF, Schafer AI. Leukotriene B4 stimulates polymorphonuclear leukocyte adhesion to cultured vascular endothelial cells. *J Clin Invest* 1984; **74**: 1552–1555.
18. Ford-Hutchinson AW, Bray MA, Doig MV, Shipley ME, Smith NJH. Leukotriene B, a potent chemokinetic and aggregating substance released from polymorphonuclear leukocytes. *Nature* 1980; **286**: 264–265.
19. Kuehl Jr FA, Dougherty HW, Ham EA. Interactions between prostaglandins and leukotrienes. *Biochem Pharmacol* 1984; **33**: 1–5.
20. Robinson DR. Prostaglandins and their regulation in rheumatoid inflammation. *Ann NY Acad Sci* 1979; **332**: 279–292.
21. Boot JR, Dawson W, Kitchen EA. The chemotactic activity of thromboxane B2: a possible role in inflammation. *J Physiol* 1976; **257**: 47P.
22. Jancar S, Teixeira CFP, Tavares de Lima W, Hoshikawa-Fujimura AY, Sirois P. Inhibitory effect of econazole on the release of thromboxanes. *Agents Actions* 1991; **34**: 387–392.
23. Wiles ME, Welbom R, Goldman G, Hechtman HB, Shepro D. Thromboxane-induced neutrophil adhesion to pulmonary microvascular and aortic endothelium is regulated by CD 18. *Inflammation* 1991; **15**: 181–199.
24. Rocha FAC, Jancar S, Fraga CAB, Timo-Iaria C, De Brum-Fernandes AJ. Platelet activating factor and histamine effects in synovial blood flow evaluated by laser-doppler flowmetry. *J Rheumatol* 1993; **20**: 1374–1377.
25. Fitzgerald MF, Henderson B, Higgs GA, Parente L, Pettipher ER. The levels of PAF and lyso-PAF in the joint fluids of rabbits with antigen-induced arthritis. *Br J Pharmacol* 1985; **86**: 422P.
26. Zarco P, Maestre C, Herrero-Beaumont G, Gonzalez E, Garcia-Hoyo R. Involvement of platelet-activating factor and tumour necrosis factor in the pathogenesis of joint inflammation in rabbits. *Clin Exp Immunol* 1992; **88**: 318–323.
27. Russo M, Arruda AMS, Jancar S. C5 fragments: are they important in PMN diapedesis? *Res Commun Chem Pathol Pharmacol* 1986; **52**: 361–369.
28. van Lent P, Joosten L, van den Bersselaar L, van den Berg W. Role of PMN in early cartilage destruction. *Agents Actions* 1991; **32**: 94–95.
29. Chatham WW, Swain R, Froshin Jr H, Heck LW, Miller EJ, Blackburn Jr WD. Degradation of human articular cartilage by neutrophils in synovial fluid. *Arth Rheum* 1993; **36**: 51–58.
30. Ugai K, Ziff M, Jasin HE. Interaction of polymorphonuclear leukocytes with immune complexes trapped in joint collagenous tissues. *Arth Rheum* 1979; **22**: 353–364.

ACKNOWLEDGEMENTS. The authors express their thanks to Dr Catarina F. P. Teixeira, Dr Nancy Starobinas, and Ms Eliane A. G. Mello, for the expert assistance with eicosanoid and TNF assays. We also thank the financial support from CNPq, FAPESP, and Fundos da Sociedade Brasileira de Reumatologia.

**Received 15 December 1995; accepted in revised form 18 January 1996**
